# Inhibition of RGMa alleviates symptoms in a rat model of neuromyelitis optica

**DOI:** 10.1038/s41598-017-18362-2

**Published:** 2018-01-08

**Authors:** Kana Harada, Yuki Fujita, Tatsusada Okuno, Shogo Tanabe, Yoshihisa Koyama, Hideki Mochizuki, Toshihide Yamashita

**Affiliations:** 1 Department of Molecular Neuroscience, Graduate School of Medicine, Osaka University, 2-2 Yamadaoka, Suita, Osaka 565-0871 Japan; 2 WPI Immunology Frontier Research Center, Osaka University, 2-2 Yamadaoka, Suita, Osaka 565-0871 Japan; 3 Department of Immunopathology, Research Institute for Microbial Diseases, Osaka University, 2-2 Yamadaoka, Suita, Osaka 565-0871 Japan; 4 Department of Neurology, Graduate School of Medicine, Osaka University, 2-2 Yamadaoka, Suita, Osaka 565-0871 Japan; 5 Department of Neuroscience and Cell Biology, Graduate School of Medicine, Osaka University, 2-2 Yamadaoka, Suita, Osaka 565-0871 Japan; 6 Graduate School of Frontier Biosciences, Osaka University, 2-2 Yamadaoka, Suita, Osaka 565-0871 Japan

## Abstract

Neuromyelitis optica (NMO) is an autoimmune disease associated with NMO immunoglobulin G (NMO-IgG), an antibody that selectively binds to the aquaporin-4. Here, we established a localized NMO model by injecting NMO-IgG into the spinal cord, and assessed the efficacy of treating its NMO-like symptoms by blocking repulsive guidance molecule-a (RGMa), an axon growth inhibitor. The model showed pathological features consistent with NMO. Systemic administration of humanized monoclonal anti-RGMa antibody delayed the onset and attenuated the severity of clinical symptoms. Further, it preserved astrocytes and reduced inflammatory-cell infiltration and axonal damage, suggesting that targeting RGMa is effective in treating NMO.

## Introduction

Neuromyelitis optica (NMO) is an autoimmune disease of the central nervous system (CNS) characterized by inflammatory lesions in the spinal cord and optic nerve, which can lead to acute transverse myelitis^1^. The prevalence of NMO is approximately 1–5 per 100,000 individuals^2^. And most of these patients show severe neurological dysfunction including limb weakness or acute attacks of optic neuritis leading to visual loss^1^. NMO is characterized by the presence of the serum autoantibody NMO-immunoglobulin G (IgG)^3^. NMO-IgG is detected in the majority of individuals with NMO and has high selectivity in binding the extracellular domain of aquaporin-4 (AQP4), a water channel found in astrocyte-foot processes in the brain^4^. Indeed, AQP4-IgG is the most widely recognized biomarker for NMO to date^5^. Because of its association with this disease, passive transfer of NMO-IgG has been used to develop rodent models of NMO^6,7^.

Because both NMO and multiple sclerosis (MS) are immune-mediated neurodegenerative diseases with broadly comparable symptoms, they may share some underlying molecular mechanisms. Recently, repulsive guidance molecule-a (RGMa) has been identified as an immune-system related protein associated with MS^8^. RGMa is upregulated around lesions after injuries to the CNS in both rats and humans^9,10^. Inhibiting RGMa with its antibody has been shown to promote axon growth and motor recovery after spinal cord injury in rats^9^, as well as improve disease scores, enhance axon growth, and reduce immune-cell invasion in the CNS of commonly used mouse and rat models of MS (i.e., experimental autoimmune encephalomyelitis)^11,12^. Recent work shows that interleukin 17A-expressing CD4^+^ T cells (Th17 cells) strongly express RGMa, and that Th17 cells induce neuronal cell death via RGMa-neogenin^13^, further support a role for RGMa in immune regulation and disease.

These findings prompted us to study whether inhibiting RGMa is also effective in treating NMO. The previous animal model of NMO generally produces disseminated lesions and causes damage to multiple neuronal circuits^14,15^, making it difficult to determine if the clinical deficits can be directly caused by a defined neuronal tract system. Here, we establish a localized model of NMO that displays the characteristic motor deficit. This procedure produces a single well-demarcated inflammatory lesion on the dorsal side of the spinal cord. The results suggest that humanized anti-RGMa monoclonal antibody (mAb) may help in preventing and attenuating the neurological symptoms of NMO.

## Results

### Establishment of a new rat model of NMO

**Figure 1:**
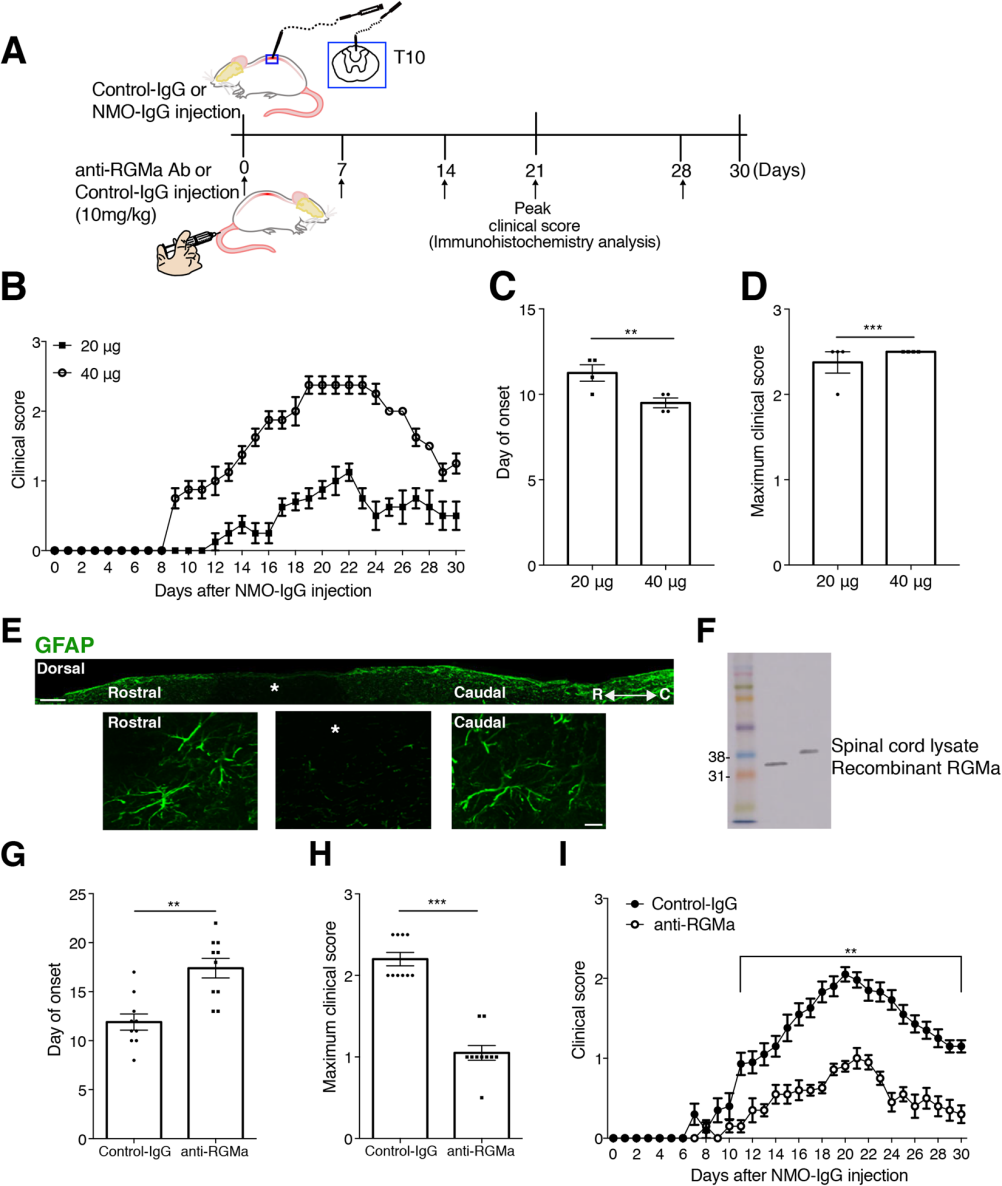
Anti-RGMa antibody alleviates symptoms in a rat model of NMO. (**A**) Schematic of the experimental procedures. A micro-syringe attached to a pulled micro-capillary needle was inserted 1 mm into the spinal cord and NMO-IgG 20 μg (human IgG from NMO patients) was infused at spinal level T10 (10th thoracic vertebrae). Arrows represent treatment days. (**B**) Comparative dose efficacy of NMO-IgG injection. Both 20 μg and 40 μg NMO-IgG treatment induces pathological features. *n* = 4. (**C**) Mean onset of symptoms and (**D**) maximum clinical score. (**E**) Longitudinal spinal cord section from the NMO-injected rat. Note the lack of GFAP expression only within the lesion site (*). Scale bars: 1 mm for the low-magnification images; 50 μm for the high-magnification images. (**F**) Specificity of anti-RGMa Ab was confirmed by western blot. (**G**–**I**) Average onset day (**G**) and average maximum clinical score (**H**) for NMO model rats given Control IgG or anti-RGMa Ab. Clinical disease score in NMO-IgG injected rats treated with either Control-IgG or anti-RGMa Ab (**I**). *n* = 10 Statistical analyses were performed using two-way ANOVA followed by Bonferroni test for B and I, and an unpaired two-tailed *t* test for C, D, G, and H. ns = not significant, ^*^ *p* < 0.05 ^**^ *p* < 0.01, ^***^ *p* < 0.001. Error bars represent the SEM.

As the first step in producing our rat model of NMO, we injected the NMO-IgG into 10th thoracic vertebrae (T10) of Wistar rats (Fig. 1A). Injection of NMO-IgG (20 μg or 40 μg) revealed neurological deficits that we presume resulted from disruption of the motor circuit (Fig. 1B), although injection of Control-IgG showed no apparent deficit (data not shown). Rats with different dosages of NMO-IgG demonstrated a dose-dependent day of onset (20 μg: 11.25 ± 0.49; 40 μg: 9.5 ± 0.29; *p* = 0.0203; *n* = 4; Fig. 1C) but no difference in peak clinical score (20 μg: 2.5 ± 0; 40 μg: 2.38 ± 0.13, *p* = 0.3559; *n* = 4; Fig. 1D). A single 20-μg NMO injection induced a well-demarcated single inflammatory lesion in the spinal cord (Fig. 1E); thus, we consider this model appropriate for modeling the development of human NMO.

### Inhibition of RGMa ameliorates the severity of NMO in model rats

For further tests, we chose to use 20-μg NMO-IgG injections (Fig. 1E–I) because low dose treatment could avoid unexpected side effects and reduce the costs of therapy. We confirmed the specificity of anti-RGMa mAb by western blot analysis using spinal cord lysate (Fig. 1F). In the NMO rats that did not receive anti-RGMa mAb, disease onset occurred after 11.9 ± 0.82 days (Fig. 1G), with the disease progressing to hind limb paresis and a mean maximum clinical score of 2.2 ± 0.08 (Fig. 1H). Compared to these rats, symptom onset for the NMO model rats that received anti-RGMa mAb was significantly later (day 17.67 ± 1.08; *p* = 0.005; Fig. 1G) and the mean peak clinical score was significantly lower (1.05 ± 0.09; *p* < 0.0001; Fig. 1H), indicating facilitated recovery. Importantly, weekly injections of anti-RGMa mAb significantly attenuated the severity of the clinical signs after day 12 (Fig. 1I).

### Anti-RGMa Ab partially restores AQP4 and GFAP expression in NMO model rats

**Figure 2:**
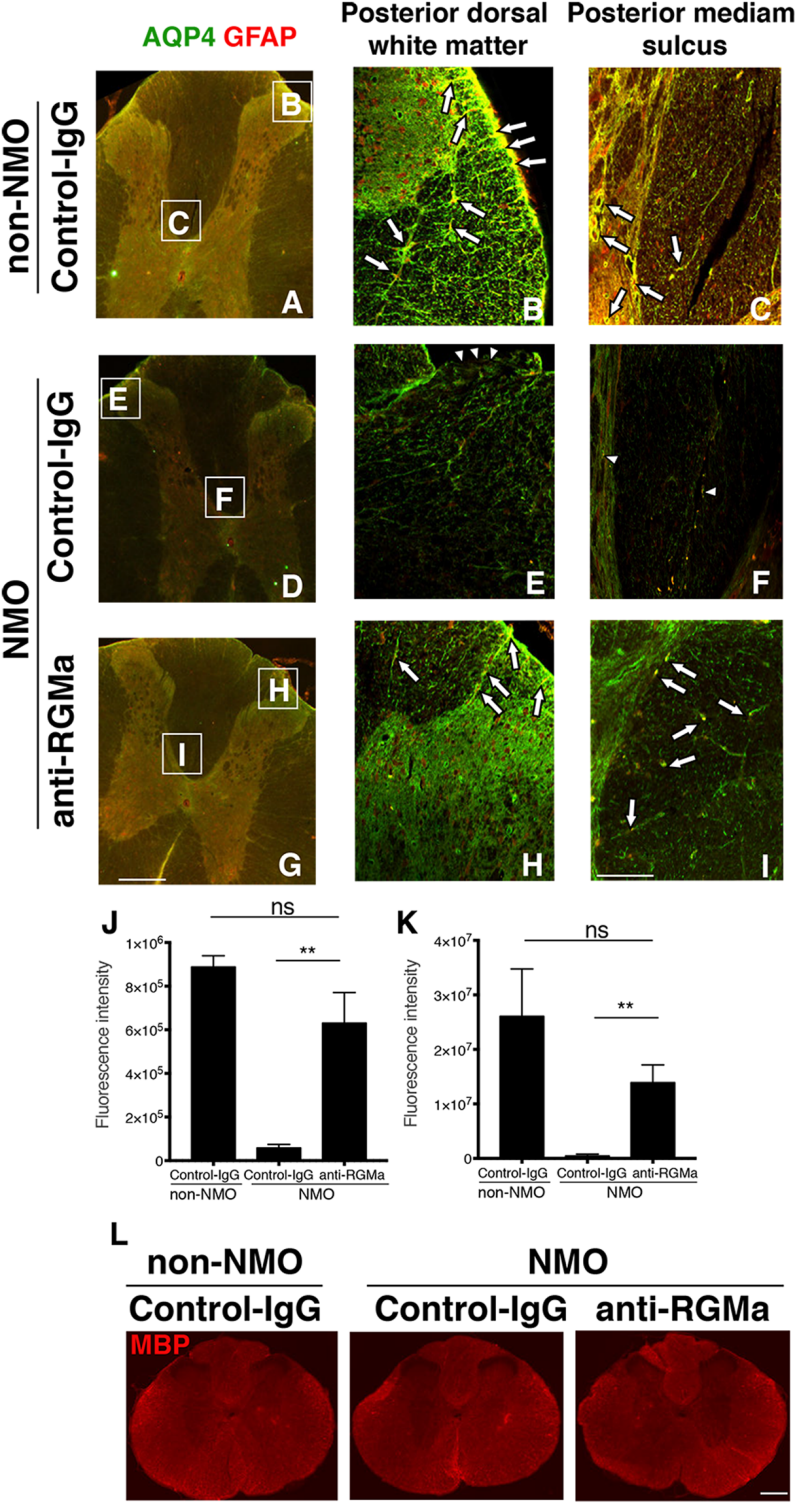
Anti-RGMa antibody ameliorates the loss of astrocyte in a rat model of NMO. (**A**–**K**) Colocalization of AQP4 and GFAP at the thoracic level of rat spinal cord tissue. (**A**,**D**,**G**). Representative fluorescence-microscopy images. Boxes are expanded in (**B**,**C**,**E**,**F**,**H**, and **I**). (**A**–**C**) Double labeling of AQP4 and GFAP was observed along the entire glia limitans, astrocytic processes, and end-feet (arrows in B), around capillaries and encircling of the blood vessels (arrows in C) in non-NMO control rats. (**D**–**F**) Co-expression of AQP4 and GFAP was lost in NMO rats that did not receive anti-RGMa mAb. Arrowheads represent the loss of co-staining along glia limitans I. Colocalization around capillaries and encircling of the blood vessels in non-NMO rats (arrows in C) was virtually absent in control-IgG-NMO rats (**F**). The arrowheads in F indicate expression of the few remaining astrocytes. (**G**–**I**) AQP4 and GFAP double-positive cells were increased in NMO rats treated with anti-RGMa mAb as compared to Control-IgG-treated NMO rats. Arrows indicate the double staining of AQP4 and GFAP in the dorsal white matter (**H**), astrocyte processes, and endfeet (**I**). (**J**,**K**) Quantitative analysis of fluorescence intensity of AQP4-(J) and GFAP- (**K**) positive cells from the high magnification images (**B**,**C**,**E**,**F**,**H**,**I**). Statistical analysis was performed by ANOVA followed by Bonferroni test for J and K. ns = not significant, ^*^ *p* < 0.05, ^**^ *p* < 0.01. Error bars represent SEM. Scale bars: 200 μm (low magnification images in **A**,**D**,**G**), 100 μm (high magnification images in (**B**,**C**,**E**,**F**,**H**,**I**). (**L**) Representative images of MBP expression, a marker for myelin. MBP expression did not display any obvious differences among the groups. Scale bar: 500 μm.

Previous reports demonstrated that lesion sites of human NMO show the loss of AQP4 and GFAP^16,17^. We therefore assessed whether anti-RGMa mAb could ameliorate the decreased distribution of AQP4 and GFAP in the lesion sites of rat NMO model. The spinal cord tissues were prepared 21 days after NMO induction. Strong AQP4 expression was observed in the white and gray matter of the non-NMO rats (Fig. 2A–C), consistent with previous results^7^. Intense AQP4 immunoreactivity was found in GFAP-positive astrocytes in the edge of white matter (Fig. 2B), and the end-feet surrounding the blood vessels (Fig. 2C) of these rats. In contrast, a massive loss of AQP4 and GFAP expression was observed in NMO rats (Fig. 2D–F). The decreased co-localization of AQP4 and GFAP in Control-IgG-treated NMO rats was restored in anti-RGMa mAb treated-NMO rats (Fig. 2G–I). Quantification analysis revealed that signal intensity of AQP4 and GFAP double-positive area was increased in NMO rats receiving anti-RGMa mAb treatment compared with Control-IgG-treated NMO rats (Fig. 2J,K). Furthermore, we examined demyelination in NMO rats. However, our rat model of NMO had no obvious loss of myelin basic protein (MBP)-immunofluorescence when compared to intact rats (Fig. 2L). This observation is consistent with the findings of a previous study^18^. Taken together, these results suggest that anti-RGMa mAb treatment prevents the loss of astrocytes in the spinal cord, which corresponds to the delay and attenuated clinical symptoms observed in the NMO-model rats.

### Anti-RGMa mAb reduces immune responses in NMO rats

**Figure 3:**
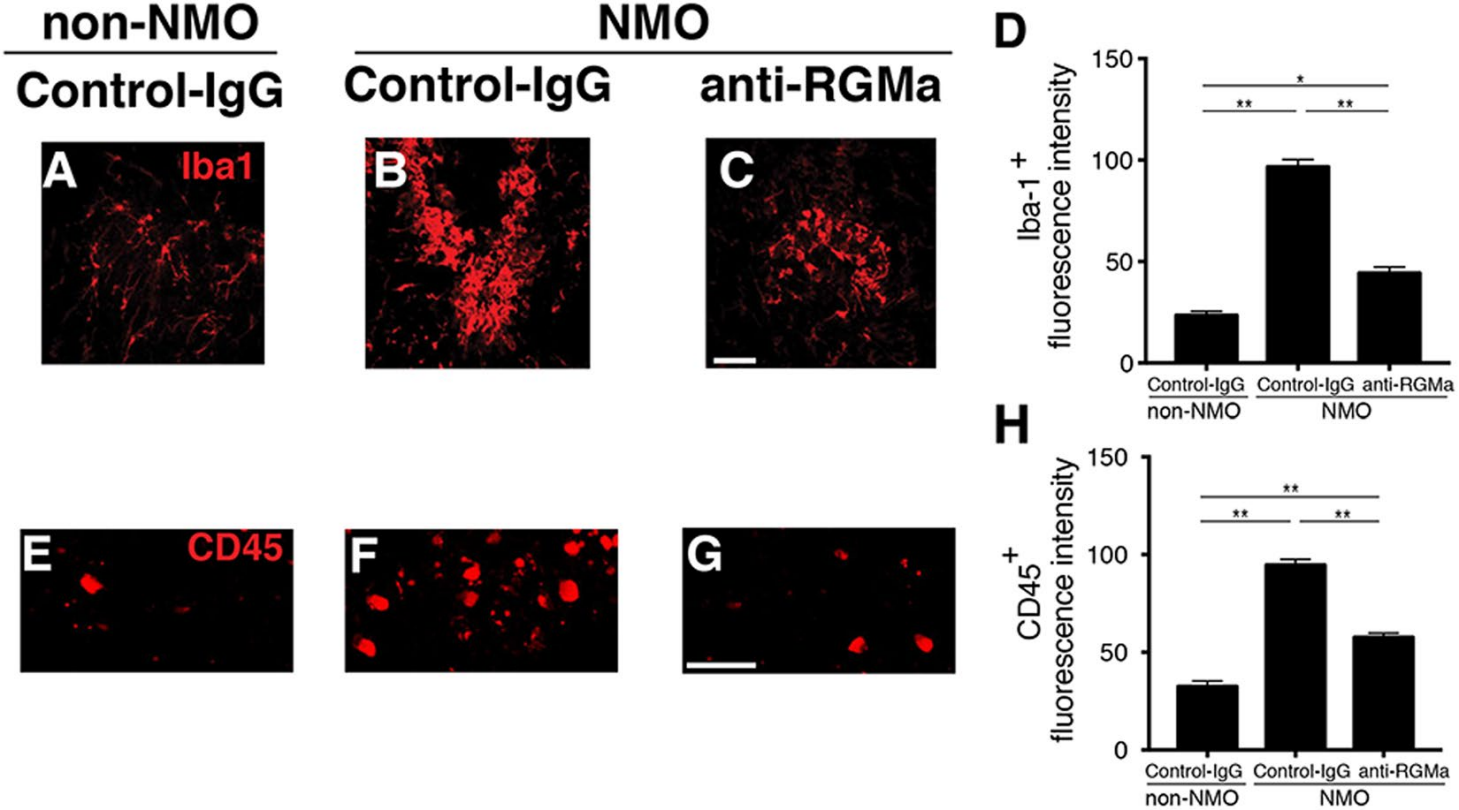
Cellular infiltration of Iba1^+^ and CD45^+^ cells in NMO rats. (**A**–**H**) Microscopic images of fluorescence intensity in rat spinal cord sections at the site of injury in order to show the distribution of Iba1^+^ cells (**A**–**C**) or CD45^+^ cells (**E**–**G**). Quantitative analysis of fluorescence intensity showed that anti-RGMa reduced the infiltration of Iba1^+^ cells (**D**) and CD45^+^ cells (**H**) at the injury site. Statistical analysis was performed by ANOVA followed by the Bonferroni test. ^*^ *p* < 0.05, ^**^ *p* < 0.01. Error bars represent SEM. Scale bars: 100 μm (**A**–**C**), 50 μm (**D**–**F**).

**Figure 4:**
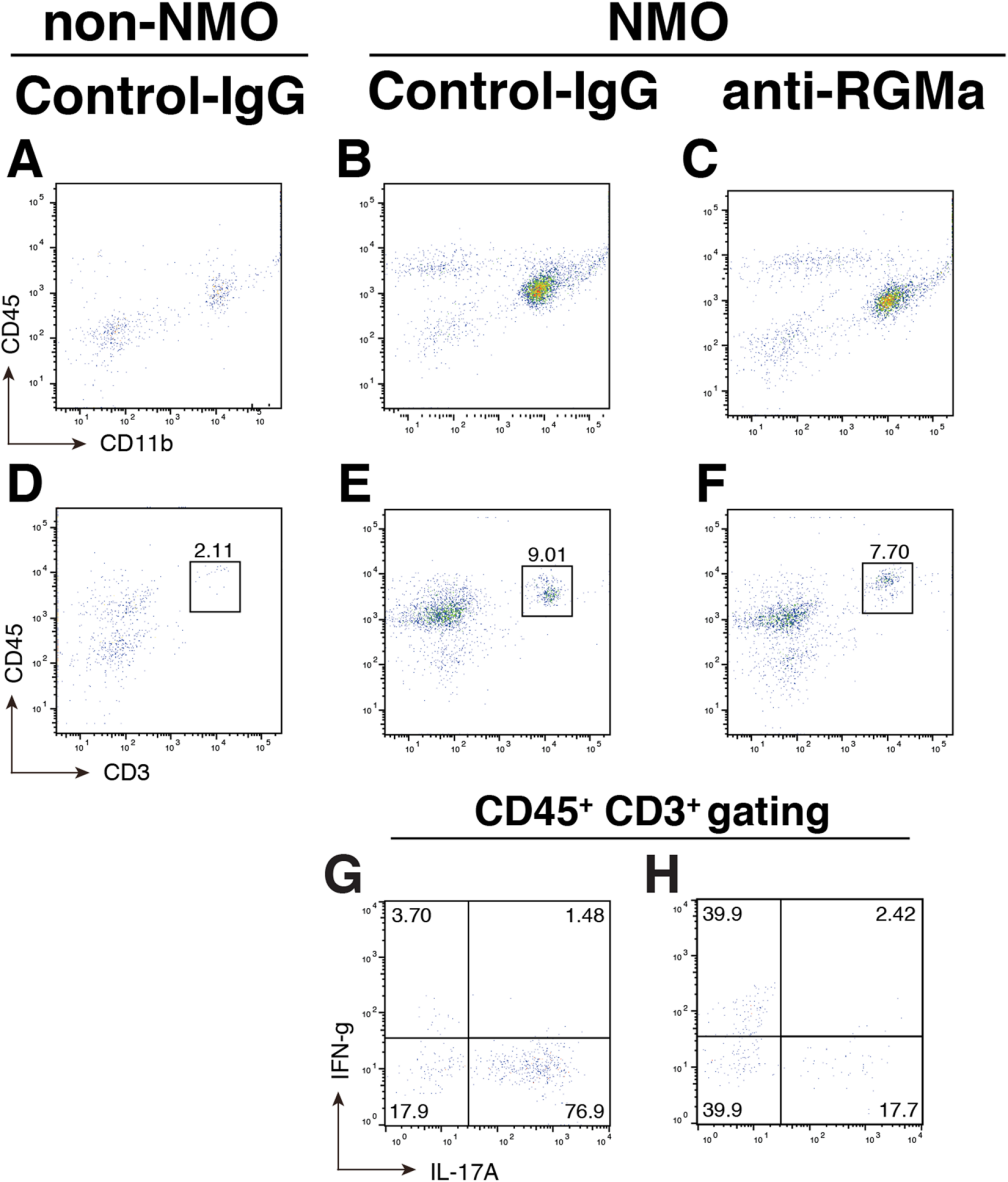
Anti-RGMa antibody reduces IL17A^+^ T-cell infiltration in a rat model of NMO. (**A**–**C**) Flow cytometry sorting of CD11b^+^ CD45^low^ (microglia) and CD11b^+^ CD45^hi^ (macrophage) from rat spinal cords at injury sites. In all groups, microglia were detected, but very few or no macrophages were detected. (**D**–**H**) Flow cytometry gating strategy of T-cells (CD3^+^, CD45^+^; **D**–**F**). Higher IL17A^+^ T-cell density was observed in Control-IgG-treated NMO rats (**G**) than in NMO rats injected with anti-RGMa mAb (**H**).

Further, because NMO-IgG-treated rats accumulated ionizing calcium-binding adaptor molecule (Iba1)-positive microglia and macrophages around their injury sites^14^, we also analyzed the immunoreactivity of Iba1 (Fig. 3A–D) and CD45 (Fig. 3E–H). Iba1^+^ cells displayed resting state (ramified) morphology in non-NMO rats (Fig. 3A). The abundance of activated cells (amoeboid morphological feature) in NMO rats without the antibody treatment (Fig. 3B) was much more than that in the NMO rats treated with anti-RGMa mAb (Fig. 3C,D). To identify these infiltrating cells, FACS sorting was used to distinguish microglia from macrophage/neutrophil. This CD11b^+^ CD45 population, defined by microglia, was detected in all three groups (Fig. 4A–C). Surprisingly, in NMO rats, there was a low number of CD11b^+^ CD45^hi^ macrophages (Fig. 4B,C). Thus, it is likely that the immunochemically labeled Iba1^+^ cells were microglia and that anti-RGMa mAb treatment reduced the accumulation of activated microglia in NMO rats. Moreover, the lateral column of spinal cord white matter in the Control-IgG injected-NMO rats displayed a strong accumulation of leukocyte common antigen CD45 (Fig. 3F). Cell infiltration in NMO rats receiving anti-RGMa mAb treatment was less (Fig. 3G), albeit still more than the very low levels of cell infiltrations in the non-NMO control rats (Fig. 3E,H). The loss of normal astrocyte function might lead to the continual entry of infiltrating cells that we observed in the NMO rats.

### RGMa inhibition attenuates the infiltration of IL-17A^+^ T-cells

Activation of T-cells is involved in NMO pathogenesis^2^. To obtain a deeper insight into the mechanism of the alleviated symptoms after inhibition of RGMa in NMO rats, we investigated the effect of RGMa inhibition on IL-17A^+^ T-cells in this NMO model. Flow cytometry (FACS) analysis of freshly isolated CD3^+^ CD45^+^ T-cells from spinal cord tissue of non-NMO and NMO rats with or without anti-RGMa mAb was evaluated (Fig. 4D–F). In non-NMO rats, low cell counts (2.11) of CD3^+^ T-cells (Fig. 4D) were detected. Further, the density (7.70) of T-cells in anti-RGMa mAb-treated NMO rats (Fig. 4C) was lower than that of NMO rats without the treatment (9.01; Fig. 4B). These results suggest that the high level of CD45 expression in control-IgG-NMO rats (Fig. 3F) may be partially due to T-cell infiltration (Fig. 4E). Notably, a high population (76.9) of IL-17A^+^ T-cells (CD3^+^, CD45^+^; Fig. 4G) was detected in NMO rats without the treatment, but this population was lower (17.7) following the anti-RGMa mAb treatment (Fig. 4H). The data imply that the inhibitory effect of anti-RGMa mAb treatment on IL-17A^+^ T-cell infiltration might contribute to the delayed onset and/or the progression of NMO in the rat model of NMO.

### RGMa inhibition attenuates neuronal damage in NMO model rats

**Figure 5:**
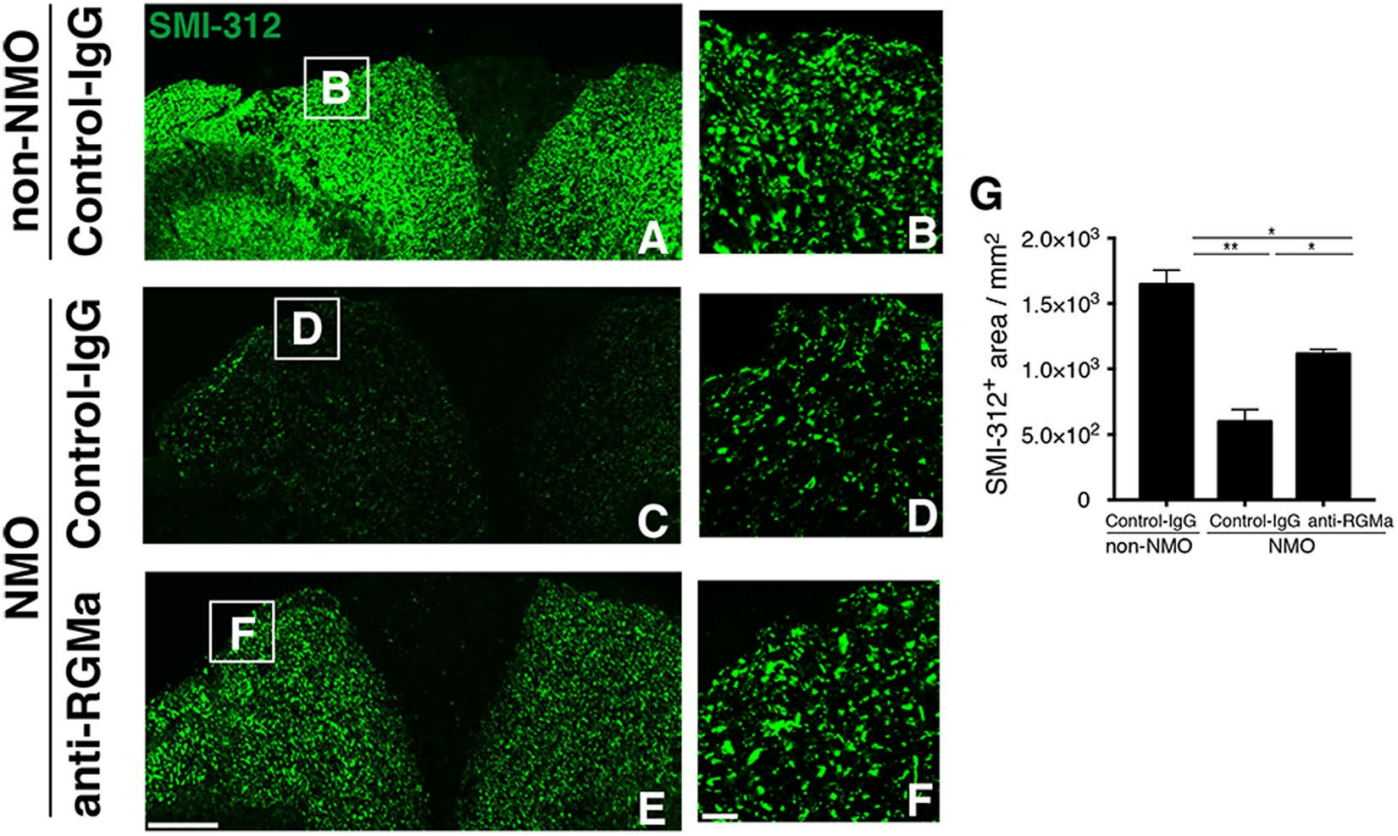
Anti-RGMa antibody reduces axonal loss in a rat model of NMO. (**A**–**F**) Representative confocal images of SMI-312-positive axonal staining in the dorsal column of rat spinal cords. Boxes are expanded in (**B**,**D**, and **F**). Number of axons was significantly less in Control-IgG-NMO rats (**C**,**D**) when compared to non-NMO rats (**A**,**B**). However, SMI-312 labeled axons were preserved in the NMO rats that received anti-RGMa mAb (**E**,**F**). (**G**) Quantitative analysis of preserved axonal counts (SMI-312^+^ axons/mm^2^) from A, C, and E (*n* = 3). Statistical analysis was performed using ANOVA followed by the Bonferroni test. ^*^ *p* < 0.05, ^**^ *p* < 0.01. Error bars represent SEM. Scale bars: 150 μm for low-magnification images (**A**,**C**,**E**,); 20 μm for high-magnification images (**B**).

A previous finding suggests that axon damage is an early feature of NMO pathology^16^. Also, in EAE mice, depletion of RGMa attenuates axonal degeneration^13^. Considering these findings, we stained the sections with a pan-axonal neurofilament marker anti-SMI-312 antibody, which specifically labels axons, to evaluate whether anti-RGMa mAb preserves axons of NMO rat spinal cords. Axons were distributed evenly throughout the dorsal column in the non-NMO rats (Fig. 5A,B). Moreover, there was a significant loss of SMI-312 expression in the control-NMO (Fig. 5C,D) when compared to the NMO rats receiving RGMa mAb injections (Fig. 5E–G). These results suggest that anti-RGMa mAb treatment presumably prevents amelioration of motor deficit in axons of our NMO rats.

## Discussion

This study established a rat model that reproduced pathological characteristics consistent with human NMO and showed that the anti-RGMa mAb prevented disease progression.

In other NMO models, rat brains were lesioned and rats received intraperitoneal injections of NMO-IgG (1 mg) for consecutive days^14^. In contrast, rats in our model received a single 20-μg injection of NMO-IgG in the dorsal column of the thoracic spinal cord, which induced a well-demarcated single inflammatory lesion in the spinal cord (Fig. 1E). This model enabled us to more clearly correlate the lesion with the deficit in motor function disrupting the corticospinal tract, the main regulator of voluntary movement. Therefore, we can determine more precisely the neuronal tract disruption by assessing the EAE score. Indeed, our NMO model rats exhibited paresis of hind limbs (Fig. 1G) accompanied by the loss of astrocytes (Fig. 2D–F), axon damage (Fig. 5C,D), high density of IL17-A^+^ T-cell infiltration (Fig. 4G), and the accumulation of activated microglia (Fig. 3B) into the thoracic spinal cord, which resemble the pathological features of human NMO^16,17,19^. In a mouse model of NMO, the loss of GFAP and AQP4 signals have been reported to occur before demyelination^18^. Hence, our model might represent an early stage of NMO, which explains the absence of demyelination (Fig. 2L), as well as why astrocytes were preserved at a wider distance in NMO rats that received anti-RGMa mAb (Fig. 2G–I).

Recently, a role for RGMa in the immune system has become apparent^8,11–13^. Bone marrow-derived dendritic cells express RGMa, which has been shown to bind to neogenin on the surface of CD4^+^ T-lymphocytes^12^. Binding RGMa to neogenin induces activation of the small GTPase Rap1, thereby increasing adhesion to intracellular adhesion molecule-1^12^. An antibody blocking RGMa was able to improve disease scores in commonly used mouse models of MS, and reduce inflammatory cell invasion into mouse CNS. It also lessened T cell proliferation and cytokine production in a mouse model of MS and in isolated peripheral blood mononuclear cells from individuals with MS^12^. Further, systemically treating rat models of experimental autoimmune encephalomyelitis with RGMa-specific antibodies significantly improved function, reduced microglial lesion size, enhanced axon regeneration into the lesion, and produced signs of remyelination^11^.

As the presence of active T-cells is considered an important factor in the progression of NMO^2^, we examined the involvement of RGMa in T-cells in order to understand the mechanism of alleviated symptoms observed in our NMO rat. Interestingly, anti-RGMa mAb-treated NMO rats displayed a sharp reduction in the IL17A^+^ T-cell population (Fig. 4H). The treatment might have brought about this effect by suppressing the enhanced production of IL17A^+^ T-cells, which subsequently reduced axonal and astrocyte loss. Furthermore, the inhibition of RGMa promotes restoration of injured neural networks, presumably leading to a delay in the progression of the secondary phase of NMO.

In summary, we propose that humanized anti-RGMa mAb might be a valid therapeutic approach that may result in attenuated pathological features of preliminary human NMO.

## Materials and Methods

### NMO-IgG preparation

Human IgGs were purified from individuals with NMO who tested positive for AQP4-Ab^7^. Informed consent was obtained from all individuals and/or their legal guardians. All methods involving humans were performed in accordance with the relevant guidelines and regulations of the ethical committees of Osaka University Hospital. All experimental protocols involving humans were approved by the ethical committees of Osaka University Hospital (11298–9).

### Surgical procedures

Wistar rats (age: 8 weeks; body weight: 200–250 g) purchased from SLC (Tokyo, Japan) were used in the experiments. Rats were anesthetized with isoflurane inhalation, and a laminectomy was performed at the thoracic 9/10 (T9/T10) vertebral level where the spinal cord was exposed. A micro-syringe (Hamilton) was attached to a pulled-glass micro-capillary needle, inserted 1 mm, and used to infuse NMO-IgG ^7^ or Control-IgG (20 μg, except where otherwise noted) at T10. Rats were randomly and evenly divided into three groups (*n* = 10 per group): non-NMO rats (control-IgG-injected rats with no treatment), Control mAb-treated NMO model rats, and anti-RGMa mAb-treated NMO model rats. For the NMO group with the treatment, we used the humanized anti-RGMa mAb (developed by Mitsubishi-Tanabe Pharma Co., Osaka, Japan), which neutralizes the effect of RGMa. Anti-RGMa mAb or Control-IgG (Mitsubishi-Tanabe Pharma Co) was injected intravenously (10 mg/kg) after the NMO-IgG injection within the spinal cord (day0) and then every 7 days (Fig. 1A). All procedures complied with the Osaka University Medical School Guidelines for the Care and Use of Laboratory Animals. All experimental protocols involving animals were approved by the institutional committee of Osaka University.

### Clinical evaluation

The severity of NMO in the rats was evaluated using clinical score as described previously^20^. 0: no symptoms; 1: flaccid tail; 2: hind limb paresis; 3: hind limb plegia, complete dragging of hind limb; 4: forelimb paresis; 5: forelimb plegia or moribund; 6: death. Appearance of a flaccid tail was considered disease onset^20^.

### Western blot

Rat spinal cords were lysed using 150 mM NaCl, 1% Triton X-100, 20 mM HEPES (pH 7.4), 10% glycerol, 5 mM EDTA, and complete protease inhibitor cocktail (Roche Applied Science). The lysates were clarified by centrifugation at 15,000 *g* at 4 °C for 30 min, and the supernatants were collected and subjected to SDS-PAGE. They were subsequently transferred onto polyvinylidene fluoride (PVDF) membranes (Millipore). The membranes were blocked with 5% bovine serum albumin in PBS containing 0.05% Tween-20 for 1 h and incubated with anti-RGMa antibody in blocking solution. After washing, the membranes were incubated with a horseradish peroxidase-conjugated secondary antibody (1: 3,000, Cell Signaling Technology) for 1 h. Detection was performed using Pierce Western Blotting Substrate Plus (Pierce) and RAS-3000 (Fuji Film).

### Immunohistochemistry

Non-NMO, control-IgG-treated NMO, and anti-RGMa mAb-treated NMO rats were transcardially perfused using 4% PFA on day 21. Immunohistochemistry was performed using primary antibodies that marked cell type, including anti-AQP4 (1:50, Santa Cruz), anti-CD45 (1:20, BD) as a pan-leukocyte marker, anti- glial fibrillary acidic protein (GFAP; 1:100; Sigma-Aldrich) as a marker for astrocytes, anti-Iba1 (1:1000, Wako) as a marker for microglia and macrophages, anti- myelin basic protein (MBP; 1:500; Dako), and anti-SMI-312 (1:1000; Covance). Images were acquired using a FV-1200 laser-scanning confocal microscope or a BX51 fluorescence microscope (Olympus). The signal intensity of AQP4 and GFAP double-positive area was quantified by setting the threshold with Image J software (NIH). The number of preserved axons in the dorsal columns of the spinal cord were counted using Image J software (NIH).

### Flow cytometry analysis

All rats were transcardially perfused using ice-cold PBS on day 21, and dissections of their spinal cord lesion sites were performed. Tissues were minced with a scalpel and digested with 0.1% collagenase D (Roche Applied Science) containing 2.5 mM calcium chloride at 37 °C for 30 min. After trituration, the cells were resuspended in 30% Percoll (GE Healthcare), under which 70% Percoll was layered, and centrifuged at 770 g for 30 min at room temperature. We isolated the cells from the interface of the 30%/70% Percoll gradients. Cells were treated with an FcR blocking reagent (Milteny Biotec) on ice for 10 min, and stained with fluorescent-conjugated antibodies: PerCP/Cy5.5 anti-rat CD45 (1:100, OX-1; Biolegend), PE anti-rat CD11b/c (1:100, OX-42; Biolegend), and APC anti-rat CD3 (1:100, 1F4; Biolegend). For intracellular cytokine staining, cells were stimulated with PMA (phorbol 12-myristate 13-acetate, 100 ng/mL, Sigma-Aldrich), ionomycin (750 ng/mL, Calbiochem), and Brefeldin A (1 *μ*g /mL, Sigma-Aldrich) for 4 h at 37 °C. Cells were subsequently treated with Fixation/Permeabilization solution (BD Biosciences), and stained with FITC anti-rat IFN-γ (1:100, DB-1; Biolegend), PE anti-IL-17A (1:100, eBio17B7; Thermo Fisher Scientific), PerCP/Cy5.5 anti-rat CD45, and APC anti-rat CD3 on ice for 30 min. We collected the data with a FACSVerse fluidics system (BD Biosciences) and analyzed it with FlowJo software (Tree Star).

### Statistical analysis

Data are presented as the mean ± SEM (standard error of the mean). Comparisons between groups were performed either by two-way ANOVA followed by post-hoc Bonferroni test or by unpaired two-tailed *t* test. Statistical significance was set at *p* < 0.05. Graphpad Prism 7 was used for the statistical analyses.

## References

[1] Wingerchuk DM, Lennon VA, Lucchinetti CF, Pittock SJ, Weinshenker BG (2007). The spectrum of neuromyelitis optica. Lancet Neurol.

[2] Flanagan, E. P. *et al*. Epidemiology of aquaporin-4 autoimmunity and neuromyelitis optica spectrum. *Ann Neurol*, 10.1002/ana.24617 (2016). 10.1002/ana.24617 PMC4988933 26891082

[3] Lennon VA (2004). A serum autoantibody marker of neuromyelitis optica: distinction from multiple sclerosis. Lancet.

[4] Lennon VA, Kryzer TJ, Pittock SJ, Verkman AS, Hinson SR (2005). IgG marker of optic-spinal multiple sclerosis binds to the aquaporin-4 water channel. J Exp Med.

[5] Tait MJ, Saadoun S, Bell BA, Papadopoulos MC (2008). Water movements in the brain: role of aquaporins. Trends Neurosci.

[6] Bradl M (2009). Neuromyelitis optica: pathogenicity of patient immunoglobulin *in vivo*. Ann Neurol.

[7] Kinoshita M (2009). Neuromyelitis optica: Passive transfer to rats by human immunoglobulin. Biochem Biophys Res Commun.

[8] Nohra R (2010). RGMA and IL21R show association with experimental inflammation and multiple sclerosis. Genes Immun.

[9] Hata K (2006). RGMa inhibition promotes axonal growth and recovery after spinal cord injury. J Cell Biol.

[10] Schwab JM (2005). Central nervous system injury-induced repulsive guidance molecule expression in the adult human brain. Arch Neurol.

[11] Demicheva E (2015). Targeting repulsive guidance molecule A to promote regeneration and neuroprotection in multiple sclerosis. Cell Rep.

[12] Muramatsu R (2011). RGMa modulates T cell responses and is involved in autoimmune encephalomyelitis. Nat Med.

[13] Tanabe S, Yamashita T (2014). Repulsive guidance molecule-a is involved in Th17-cell-induced neurodegeneration in autoimmune encephalomyelitis. Cell Rep.

[14] Asavapanumas N, Verkman AS (2014). Neuromyelitis optica pathology in rats following intraperitoneal injection of NMO-IgG and intracerebral needle injury. Acta Neuropathol Commun.

[15] Saadoun S (2010). Intra-cerebral injection of neuromyelitis optica immunoglobulin G and human complement produces neuromyelitis optica lesions in mice. Brain.

[16] Lucchinetti CF (2002). A role for humoral mechanisms in the pathogenesis of Devic’s neuromyelitis optica. Brain.

[17] Misu T (2007). Loss of aquaporin 4 in lesions of neuromyelitis optica: distinction from multiple sclerosis. Brain.

[18] Zhang H, Verkman AS (2013). Eosinophil pathogenicity mechanisms and therapeutics in neuromyelitis optica. J Clin Invest.

[19] Popescu BF (2011). Neuromyelitis optica unique area postrema lesions: nausea, vomiting, and pathogenic implications. Neurology.

[20] Kurosawa K (2014). Severely exacerbated neuromyelitis optica rat model with extensive astrocytopathy by high affinity anti-aquaporin-4 monoclonal antibody. Acta Neuropathol Commun.

